# PET/CT Imaging with an ^18^F-Labeled Galactodendritic Unit in a Galectin-1–Overexpressing Orthotopic Bladder Cancer Model

**DOI:** 10.2967/jnumed.119.236430

**Published:** 2020-09

**Authors:** Patricia M.R. Pereira, Sheryl Roberts, Flávio Figueira, João P.C. Tomé, Thomas Reiner, Jason S. Lewis

**Affiliations:** 1 Department of Radiology, Memorial Sloan Kettering Cancer Center, New York, New York; 2 QOPNA and LAQV-REQUIMTE, Department of Chemistry, University of Aveiro, Aveiro, Portugal; 3 CICECO, Departamento de Química, Universidade de Aveiro, Aveiro, Portugal; 4 CQE and Departamento de Engenharia Química, Instituto Superior Técnico, Universidade de Lisboa, Lisboa, Portugal; 5 Department of Radiology, Weill Cornell Medical College, New York, New York; 6 Chemical Biology Program, Memorial Sloan Kettering Cancer Center, New York, New York; 7 Molecular Pharmacology Program, Memorial Sloan Kettering Cancer Center, New York, New York; 8 Department of Pharmacology, Weill Cornell Medical College, New York, New York; and; 9 Radiochemistry and Molecular Imaging Probes Core, Memorial Sloan Kettering Cancer Center, New York, New York

**Keywords:** dendritic carbohydrate, PET/CT imaging, bladder cancer, galectins, ^18^F-radiochemistry

## Abstract

Galectins are carbohydrate-binding proteins overexpressed in bladder cancer (BCa) cells. Dendritic galactose moieties have a high affinity for galectin-expressing tumor cells. We radiolabeled a dendritic galactose carbohydrate with ^18^F (^18^F-labeled galactodendritic unit **4**) and examined its potential in imaging urothelial malignancies. **Methods:** The ^18^F-labeled first-generation galactodendritic unit **4** was obtained from its tosylate precursor. We conducted *in vivo* studies in a galectin-expressing UMUC3 orthotopic BCa model to determine the ability of ^18^F-labeled galactodendritic unit **4** to image BCa. **Results:** Intravesical administration of ^18^F-labeled galactodendritic unit **4** allowed specific accumulation of the carbohydrate radiotracer in galectin-1–overexpressing UMUC3 orthotopic tumors when imaged with PET. The ^18^F-labeled galactodendritic unit **4** was not found to accumulate in nontumor murine bladders. **Conclusion:** The ^18^F-labeled galactodendritic unit **4** and similar analogs may be clinically relevant and exploitable for PET imaging of galectin-1–overexpressing bladder tumors.

Bladder cancer (BCa) is the tenth most prevalent cancer worldwide (1). The GLOBOCAN 2018 estimated globally nearly 549,000 new BCa cases and about 200,000 deaths (1). Most BCa arises in the bladder urothelium and is classified as urothelial carcinoma—also named transitional cell carcinoma (2). Transitional cell carcinoma represents more than 70% of patient cases, and it is a noninvasive disease that commonly recurs in the urinary tract only. BCa muscle-invasive and metastatic disease, which is the lethal phenotype of BCa, occurs in nearly 20% of the patients (3).

BCa evaluation involves cystoscopy to collect a tumor sample, a procedure named transurethral resection of bladder tumor, followed by histologic confirmation (4). Transurethral resection of bladder tumor requires that the resection be deep enough to include the muscularis of the bladder in order to properly determine and stage whether the tumor is muscle-invasive or just *in situ*. Fluorescence cystoscopy and narrow-band imaging enhance cystoscopic detection of BCa because of their high specificity to the tumor tissue (5,6). CT, MRI urography, and ultrasound imaging have also been demonstrated to enhance diagnostic accuracy in patients with BCa (7). However, during tumor development, phenotypic alterations occur before morphologic modifications are evident by cystoscopy, CT, MRI, or ultrasound (8). PET does not require tumor resection and may delineate these phenotypic alterations before morphologic ones are apparent and therefore complements the use of transurethral resection of bladder tumor. Tumor-targeting vectors bound to a positron-emitting radionuclide (e.g., ^18^F) are widely used in oncology diagnostic imaging (8). In this context, the glucose analog ^18^F-FDG accumulates in tumor cells as a result of their high glucose uptake mediated by glucose transporters. Although ^18^F-FDG PET/CT allows detection of metastatic disease, renal excretion of the glucose radiotracer results in difficulty identifying primary lesions of the bladder.

Recent studies have identified a role for galectins in BCa development and progression (9–12), suggesting their potential as biomarkers and providing clinical opportunities for the development of galectin-directed imaging and therapeutic approaches (13). Galectins are a family of proteins containing carbohydrate-binding domains with strong affinity for carbohydrate structures that consist of galactose residues (14). Galectin-1 and galectin-3 are significantly upregulated in BCa compared with normal cells and contribute to tumor growth and invasion (13). In a study with 185 BCa patient samples, 75% of the specimens demonstrated that *LGALS1* (a gene that codes for galectin-1) amplification and galectin-1 protein expression predicted disease-specific survival (11). In addition, galectin-1 expression positively correlated with histologic grade and pathologic tumoral stage and could discriminate between non–muscle-invasive and muscle-invasive BCa (13). Oligomerization of galectins results in carbohydrate-binding domain clustering and allows multivalent interactions with carbohydrates, resulting in the formation of ordered arrays (galectin-carbohydrate lattices) (15). Pereira et al. previously developed a dendritic galactose unit to be conjugated with porphyrinoids (14) with high affinity for galectin-1–overexpressing BCa cells (16,17). Such a galactose dendritic molecule allows high affinity to tumor cells due to the ability of galectin-1 to establish multivalent interactions with the galactose units of the dendritic unit.

Here, we demonstrate the utility of the radiolabeled galactodendritic unit, ^18^F-labeled galactodendritic unit **4,** to image galectin-1–overexpressing UMUC3 cells that were derived from human urinary BCa and orthotopically implanted in the murine bladder.

## MATERIALS AND METHODS

Chemicals were obtained from commercial suppliers and used without further purification unless otherwise stated. 2-mercaptoethanol, tetra-*n*-butylammonium fluoride, 4-toluenesulfonyl chloride, chemical reagents (*N,N*-diisopropylethylamine, triethylamine, potassium carbonate, sodium bicarbonate, and trifluoroacetic acid), and solvents (tetrahydrofuran, dichloromethane, acetone, chloroform-*d*, and a high-performance liquid chromatography [HPLC] and liquid chromatography–mass spectrometry grade of acetonitrile) were purchased from Sigma-Aldrich. Water (18.2 MΩ cm^−1^ at 25°C) was obtained from an Alpha-Q Ultrapure water system from Millipore. HPLC purification and analysis were performed on a Shimadzu ultra-fast HPLC system with a DGU-20A degasser, an SPD-M20A ultraviolet detector, an LC-20AB pump unit, and a CBM-20A communication bus module. All HPLC purification was performed using semipreparative HPLC (Phenomenex Gemini C18, 5 μm, 10 × 250 mm, 3.5 mL/min 5%–95% water/acetonitrile 10-min linear gradient) unless otherwise stated. Liquid chromatography–mass spectrometry using electrospray ionization was performed on a Waters instrument with single-quadrupole detector for mass identification. A lyophilizer (FreeZone 2.5 Plus; Labconco) was used for freeze-drying. ^1^H nuclear MR and ^13^C nuclear MR spectra were recorded on a Bruker AV 500-MHz device at the Department of Chemistry in Aveiro, Portugal. ^1^H nuclear MR data are reported as chemical shifts in relative parts per million (δ) and referenced to residual protic peaks. The coupling constants, *J,* are quoted in hertz and its multiplicities by s (singlet), d (doublet), t (triplet), q (quartet), m (multiplet), and br (broadened). ^13^C nuclear MR data are reported in parts per million relative to the solvent. All averages are presented as mean ± SD. Chromatograms and spectra were plotted on the GraphPad software Prism, version 7.

### Synthesis

#### Preparation of Compound **1**

Compound **1** is 2-[(4,6-bis(1,2:3,4-di-*O*-isopropylidene-α-d-galactopyran-6-yl)-1,3,5-triazin-2-yl)thio]ethan-1-ol. In a 25-mL round-bottomed flask, di-galactotriazine (200.0 mg, 0.31 mmol, 1.0 equivalent) and *N,N*-diisopropylethylamine (0.17 mL, 0.95 mmol, 3.0 equivalent) were dissolved in 8 mL of dry tetrahydrofuran and air-purged with bubbling nitrogen for 15 min. The reaction was then cooled in an ice bath. 2-mercaptoethanol (32.1 mg, 0.41 mmol, 1.3 equivalent) was added carefully to the reaction. The reaction was deemed finished after 3 h, and purification on 20 × 20 cm thin-layer chromatography plates with a 0.25-mm silica developed using ethyl acetate:hexane (1:1) furnished 173.0 mg (85%) of **1** as a colorless oil. ^1^H nuclear MR (500 MHz, chloroform-*d*) δ 5.55 (d, *J* = 5.0 Hz, 2H), 4.64 (dd, *J* = 7.9, 2.5 Hz, 2H), 4.53 (d, *J* = 6.5 Hz, 4H), 4.40–4.30 (m, 4H), 4.24–4.14 (m, 2H), 3.89 (t, *J* = 6.2 Hz, 2H), 3.32 (t, *J* = 6.2 Hz, 2H), 1.52 (s, 6H), 1.46 (s, 6H), 1.35 (s, 6H), 1,33 (s, 6H). ^13^C nuclear MR (126 MHz, chloroform-*d*) δ 184.8, 170.4, 109.6, 108.8, 96.3, 70.6, 70.6, 70.5, 66.5, 65.5, 61.9, 33.0, 26.1, 26.0, 24.9, 24.4. Retention time = 13.35 min. Electrospray ionization–mass spectrometry: m/z calculated for C_29_H_43_N_3_O_13_S: 673.25; found: 674.3 [M + H]^+^.

#### Preparation of Compound **2**

Compound **2** is 2-[(4,6-bis(1,2:3,4-di-*O*-isopropylidene-α-d-galactopyran-6-yl)-1,3,5-triazin-2-yl)thio]ethan-1-oxytosylate. Compound **1 (**15 mg, 22 μmol, 1.0 equivalent**)** and 4-toluenesulfonyl chloride (13 mg, 33 μmol, 3 equivalent) were dissolved in 250 μL of dichloromethane. The solution was cooled to 0°C using an ice/acetone bath. Once cooled, a cold solution of triethylamine (12 μL, ρ = 0.726 g/cm^3^, 88 μmol, 4 equivalent) in 250 μL of dichloromethane was added dropwise. The reaction mixture was stirred at 0°C until the ice/acetone bath melted and then was stirred at room temperature overnight. The reaction mixture was dried in vacuo and then resuspended in 250 μL of acetonitrile with 5% water, and HPLC purification afforded 15.66 mg (85%) of **2.** Retention time = 9.33 min. Electrospray ionization–mass spectrometry: m/z calculated for C_36_H_49_N_3_O_15_S_2_: 827.26; found: 828.63 [M + H]^+^.

#### Preparation of Compound **3**

Compound **3** is 2-[(4,6-bis(1,2:3,4-di-*O*-isopropylidene-α-d-galactopyran-6-yl)-1,3,5-triazin-2-yl)thio]ethane-1-^19^F-fluorine. Compound **2** (1.17 mg, 1.41 μmol, 1.0 equivalent) and potassium carbonate were dissolved in 300 μL of acetonitrile before tetra-*n*-butylammonium fluoride (18.56 mg, 71 μmol, 50 equivalent) in tetrahydrofuran was added to the mixture. The reaction mixture was stirred at 90°C for 10 min and diluted with acetonitrile. HPLC purification afforded 0.67 mg (72%) of **3.** Retention time = 12.11 min. Electrospray ionization–mass spectrometry: m/z calculated for C_29_H_42_FN_3_O_12_S: 656.25; found: 656.50 [M + H]^+^.

#### Preparation of Compound **4**

Compound **4** is 2-[(4,6-bis(α/β-galactose-6-yl)-1,3,5-triazin-2-yl)thio]ethane-1-^19^F-fluorine. Compound **3** was treated with a solution of trifluoroacetic acid and dichloromethane (9:1), followed by the addition of 1 drop of water (∼200 μL). The reaction mixture was stirred at 60°C for 20 min. The reaction was neutralized with sodium bicarbonate and diluted with 500 μL of water. The resulting mixture was purified by HPLC, affording a qualitative yield (>97%) of **4.** Retention time = 3.40 min. Electrospray ionization–mass spectrometry: m/z calculated for C_17_H_26_FN_3_O_12_S: 496.12; found: 496.98 [M + H]^+^.

### Radiosynthesis

A standard procedure to separate the ^18^F-fluoride in a form that is suitable for nucleophilic fluorination was performed, as previously described in detail (18,19). Radiosynthesis of ^18^F-compound **4** and its deprotection were completed in 2 steps *in situ*, using HPLC purification of the final product **4** to ensure chemical and radiochemical purity and a molar activity of more than 0.407 GBq/μmol. ^18^F-compound **4** was prepared in dry dimethylsulfoxide (500 μL) by adding compound **3** (200 μg) to the vial used to dry the ^18^F-fluoride. The vial was sealed and heated to 90°C for 6 min under stirring. Either the solution was diluted with 1 mL of water and purified by HPLC—obtaining the radioactive compound **3** in qualitative yields greater than 85% (non–decay-corrected [n.d.c.])—or the reaction was performed *in situ* by treating the solution with trifluoroacetic acid:dichloromethane (9:1), followed by the addition of 1 pipette drop of water. The reaction was stirred for 20 min at 60°C. The reaction was then neutralized with sodium bicarbonate, diluted with 700 μL of water, and purified on HPLC. The collected peak (3.40–3.80 min) was diluted 10:1 with water, trapped on a Waters C18 Sep-Pak Light cartridge, eluted with 500 μL of ethanol, and diluted to less than 5% ethanol with sterile normal saline. ^18^F-compound **4** was obtained in 45% overall n.d.c. radiochemical yield (based on measuring the isolated final product on a dose calibrator at the end of formulation), 99% radiochemical purity after purification, and a molar activity of more than 0.407 GBq/μmol. The synthesis was performed manually, starting with 2,220 MBq (60 mCi) of ^18^F-fluoride as delivered by the cyclotron; radiochemical yield is calculated from the activity eluted from the anion exchange cartridge. The fraction collected during the first HPLC run was approximately 3 mL. Cold chemistry was performed under slightly different conditions (as described in the synthesis section). The synthesis was completed within about 1.5 half-lives from the end of bombardment.

The ^18^F-FDG was obtained from the nuclear pharmacy at Memorial Sloan Kettering Cancer Center on the morning of injection.

### BCa Cell Lines and Cell Culture

Human BCa cells UMUC3, HT1197, and T24 were obtained from the American Type Culture Collection. The UMUC14 and RT112 BCa cells were obtained from Sigma-Aldrich and European Collection of Authenticated Cell Cultures. The cells were cultured according to the recommendations of the American Type Culture Collection, Sigma-Aldrich, or the European Collection of Authenticated Cell Cultures and used within a passage number of 6.

### Galectin-1 Interaction Assays, Galectin-1 Knockdown, and Binding Studies in BCa Cells

A solution of galectin-1 (Sigma) at a concentration of 2 μM was titrated with increasing concentrations of ^19^F-compound **4** (0–3.2 μM). The fluorescence emission spectra of the tryptophan residues in the galectin-1 protein were acquired for the wavelength range between 300 and 450 nm on excitation at 280 nm. The fluorescence-quenching curves were obtained by plotting the tryptophan residues quenching (in percentage) against the ^19^F-compound **4** concentration. The dissociation constants of the galactose conjugates to galectin-1 were calculated using the Boltzmann sigmoidal model (20).

Galectin-1 was depleted in UMUC3 BCa cells using a pool of 3 target-specific 20–25 nucleotides (nt) small interfering RNA (Santa Cruz Biotechnology) following previously reported methodology (16,17).

BCa cells (0.25 × 10^6^) resuspended in 100 μL of ice-cold phosphate-buffered saline (PBS) were incubated with 0.037 MBq of ^18^F-galactodendritic unit **4** during 1 h at 4°C with gentle agitation. The cells were then centrifuged at 1,400*g* for 4 min at 4°C, and supernatant containing unbound radioactivity was collected into an Eppendorf tube labeled #1. The cells were washed twice with 1 mL of PBS and centrifuged, and supernatants were collected into an Eppendorf tube labeled #2. The pellet was resuspended in radioimmunoprecipitation assay buffer. The pellet and supernatants were measured for radioactivity on a γ-counter calibrated for ^18^F. The percentage of binding was calculated using the following formula:

$${\text{Radioactivity}}_{\text{pellet}}/\left({\text{radioactivity}}_{\text{pellet}}+{\text{radioactivity}}_{\text{Eppendorf\#}1}+{\text{ radioactivity}}_{\text{Eppendorf\#}2}\right)\times 100\text{.}$$

Protein concentration was determined using a bicinchoninic acid protein assay kit (Pierce), and the percentage of binding was then normalized to the amount of protein.

### Orthotopic BCa Model

All experiments involving animals were performed according to the guidelines approved by the Research Animal Resource Center and Institutional Animal Care and Use Committee at Memorial Sloan Kettering Cancer Center. The first author of this article has a category C accreditation for animal research given by the Federation of European Laboratory Animal Science, and in this study, we adhered to the “Animal Research: Reporting of *In Vivo* Experiments” guidelines and to the guidelines for the welfare and use of animals in cancer research.

Bladder orthotopic models were developed by inoculating UMUC3 cells into the bladder of 8- to 10-wk-old *nu/nu* female mice. The mice were purchased from Charles River Laboratories.

The mice were anesthetized by inhalation of 1%–2% isoflurane (Baxter Healthcare) in an oxygen gas mixture and kept on a heated platform during catheterization procedures. An angiocatheter (24-gauge; Terumo Medical Products) and the area of catheterization were lubricated before the catheter was inserted into the urethra. After full insertion, the catheter was lifted gently and kept parallel to the bench. To avoid periurethral leakage and reflux to the upper urinary tract, the urine was removed from the bladder by abdominal massage and by application of suction to the external end of the catheter. The bladder was flushed with 80 μL of sterile PBS and pretreated with 80 μL of poly-l-lysine (Sigma) for 15 min. A single-cell suspension of 5 × 10^5^ UMUC3 cells in 50 μL of medium was inoculated into the bladder via the urethra with the angiocatheter. To improve the tumor cell uptake, the catheter–syringe assembly was left in place for 40 min. This methodology allowed us to obtain a tumor take rate of 90%. During the entire procedure, the mice were kept under anesthesia for 2 h before the catheter–syringe assembly was gently removed from the urethra. The mice were monitored every day for any signs of pain and distress, and ultrasound imaging was used to monitor tumor development.

### Ultrasound Imaging of Bladder Tumor

At 7 and 15 d after the inoculation of UMUC3 BCa cells, the developed bladder tumors were noninvasively imaged using the Vevo 2100 Imaging System (VisualSonics). The transducer was placed in the mid-pelvic region of the animal in transverse orientation until the bladder (a large black structure) appeared in the pelvic region. The images were then acquired in B-mode: 40-MHz frequency, 100% power, 52-frame rate, 30.0-dB gain, 10-mm depth, 14.08-mm width, high line density, high sensitivity, 65-dB dynamic range, G5 display map, 50 brightness, and 50 contrast.

### PET/CT Imaging and Acute Biodistribution Studies

At 15 d after the inoculation of UMUC3 BCa cells, mice (3 per group) were either intravenously administered ^18^F-labeled galactodendritic unit **4** (2.9–3.3 MBq) or intravesically administered (i.e., instillation directly into the bladder via insertion of an urethral catheter) 14.7–15.3 MBq of ^18^F-galactodendritic unit **4** or ^18^F-FDG.

At 0.5, 1, and 2 h after intravenous administration of ^18^F-labeled galactodendritic unit **4,** PET images were recorded on an Inveon PET/CT scanner (Siemens).

At 30 min after intravesical administration, the mice were anesthetized with 1.5%–2% isoflurane, the bladder was fully emptied and flushed with PBS, and PET images were recorded (following previously reported methodology (21)) at 1 h after intravesical administration. All images were visualized in AMIDE software (version 1.0.4; http://amide.sourceforge.net).

Acute biodistribution studies were performed at 2 h after intravenous injection of ^18^F-galactodendritic unit **4** (21), and the radioactivity associated with each organ was expressed as a percentage of injected dose per gram of organ.

### ^18^F-Galactodendritic Unit 4 Stability in Saline Solution Containing 10.5% (v/v) Mouse Urine

Stability studies were performed in a saline solution containing 10.5% (v/v) mouse urine, as this mimics the *in vivo* conditions used in the PET imaging studies after the intravesical administration of the radiotracer. In the time between emptying of the mouse bladder and the start of the PET imaging, we observed 10 μL of urine excretion (corresponds to 10.5% of bladder mouse capacity, or ∼105 μL). Urine was collected from mice with UMUC3 orthotopic BCa. To 170 μL of PBS were added 740 kBq (20 μCi; 10 μL) of compound **4.** A 20-μL volume of urine was added to the solution and shaken. Stability was tested by reversed-phase HPLC with in-line radiation (Posi-RAM model 4; LabLogic) detection using a Kinetex Biphenyl column (150 × 4.6 mm; 5-μm particle size; Phenomenex) and a mobile phase gradient of 5%–20% acetonitrile (0.1% trifluoroacetic acid) in water (0.1% trifluoroacetic acid) over 20 min. Intact ^18^F-compound **4** elutes at 12 min and is 75% intact (derived from radiotracer chromatogram) or 50% intact (derived from HPLC chromatogram) after 30 min of incubation; several metabolites eluted at later time-points after 1 h (full characterization is provided in the supplemental materials, available at http://jnm.snmjournals.org). The percentages of intact radiotracer and metabolites were obtained from calculating the area under the curve.

## RESULTS

### Radiosynthesis of ^18^F-Labeled Galactodendritic Unit 4

**FIGURE 1.**
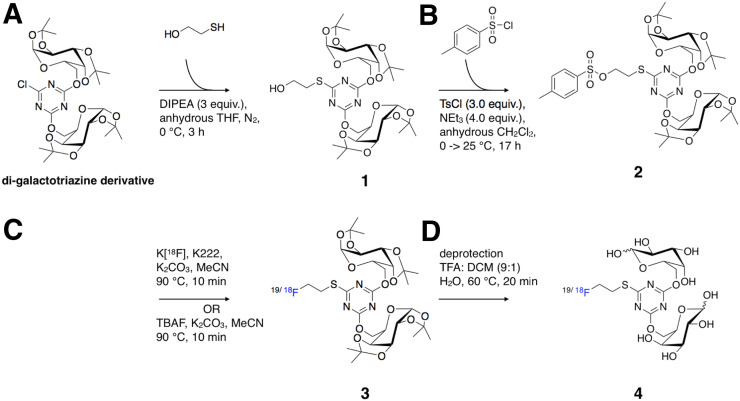
Synthetic reaction and conditions for synthesis of ^19^F/^18^F-galactodendritic unit **4.** (A) 2-mercaptoethanol, *N,N*-diisopropylethylamine (DIPEA), anhydrous tetrahydrofuran (THF), N_2_, 0°C, 3 h. (B) 4-toluenesulfonyl chloride, NEt_3_, anhydrous dichloromethane, 0°C → 25°C, 17 h. (C) Tetra-*n*-butylammonium fluoride (TBAF), K_2_CO_3_, MeCN, 90°C, 10-min optimization-of-reaction radiolabeling conditions activating K^18^F, K_222_/K_2_CO_3_, MeCN, 90°C, 10 min. (D) Trifluoroacetic acid (TFA):dichloromethane (DCM) (9:1), H_2_O, 60°C, 20 min.

In 2012, Silva et al. prepared a first-generation galactodendritic unit based on 2,4,6-trichloro-1,3,5-triazine (14). Herein, we have attempted these syntheses by changing the terminal group in the spacer region to a hydroxyl group, facilitating further manipulation (Fig. 1). The di-nucleophilic substitution of 2,4,6-trichloro-1,3,5-triazine by the galactose moiety was performed as previously reported (14). The reaction of the di-galactotriazine with 2-mercaptoethanol provided the first-generation dendritic unit **1** in about 85% yield (Supplemental Figs. 1–4). We chose to pursue the tosylation of compound **1** at its phenol position because affording compound **2** is the most facile route for subsequent nucleophilic substitution (SN_2_) of fluorine. This is especially important because the incorporation of its radioactive analog ^18^F-F allowed the shortest time for handling radioactive material. The small conversion of this group to an alkyl ether was predicted not to perturb targeting and galectin binding, as we are not altering the hydroxyl groups in the carbohydrate moiety responsible for galactose binding to galectins (14,16,17). The tosylation of compound **1** with 4-toluenesulfonyl chloride produced the desired product **2** in 85% yield. Compound **2** was reacted with tetra-*n*-butylammonium fluoride to produce ^19^F-compound **3**. The subsequent deprotection of compound **3** gave the desired product, which eluted at 3.40 min on the reverse-phase HPLC column, indicating good separation from compound **3.** We confirmed the chemical identity of all products (Supplemental Figs. 5–7).

**FIGURE 2.**
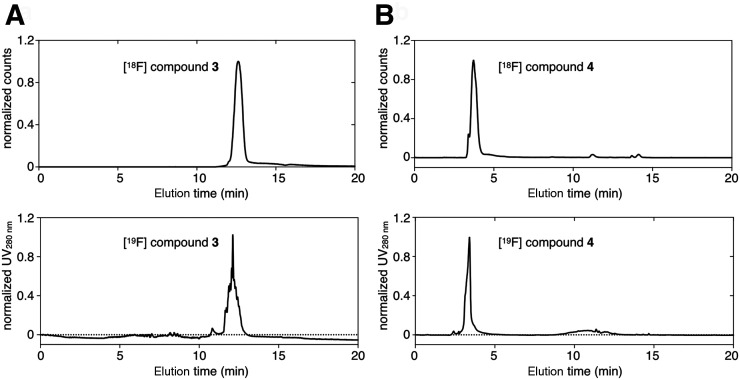
Confirmation for chemical identity of ^18^F-galactodendritic carbohydrates **3** and **4** by HPLC using verified cold standard. (A) Radio-HPLC chromatogram of ^18^F-galactodendritic carbohydrate **3** (top) and ^19^F-galactodendritic carbohydrate **3** (bottom). (B) Radio-HPLC chromatogram of ^18^F-galactodendritic unit **4** (top), coeluted with ^19^F-galactodendritic unit **4** (bottom). UV = ultraviolet.

The production of ^18^F-labeled compound **4** was completed following a well-established ^18^F-fluoroethylation, similar to previously described work (Fig. 1) (18,19). The synthesis was completed with 45% radiochemical yield (n.d.c.), 99% radiochemical purity after purification. ^18^F-labeled compound **3** could be isolated in a qualitative radiochemical yield of greater than 85% (n.d.c.) and coeluted with ^19^F-compound **3** at 12.11 min on HPLC (Fig. 2). ^18^F-compound **4** coeluted with ^19^F-compound **4** at 3.40 min on HPLC, confirming their chemical identity (Fig. 2).

### Binding of ^18^F-Labeled Galactodendritic Unit 4 to BCa Cells Containing High Levels of Galectin-1 Protein

Porphyrinoids attached to the galactodendritic unit **4** have demonstrated in previous studies the ability to target galactose-binding proteins, such as galectin-1, overexpressed in tumor tissues (16,17). The ^19^F-compound **4** interacts with galectin-1 protein (Supplemental Figs. 8 and 9), showing values of acid dissociation constant and *n* equal to 8.778 × 10^7^ M^−1^ and 1.0, respectively, as well as a dissociation constant equal to 0.067 ± 0.01 μM.

**FIGURE 3.**
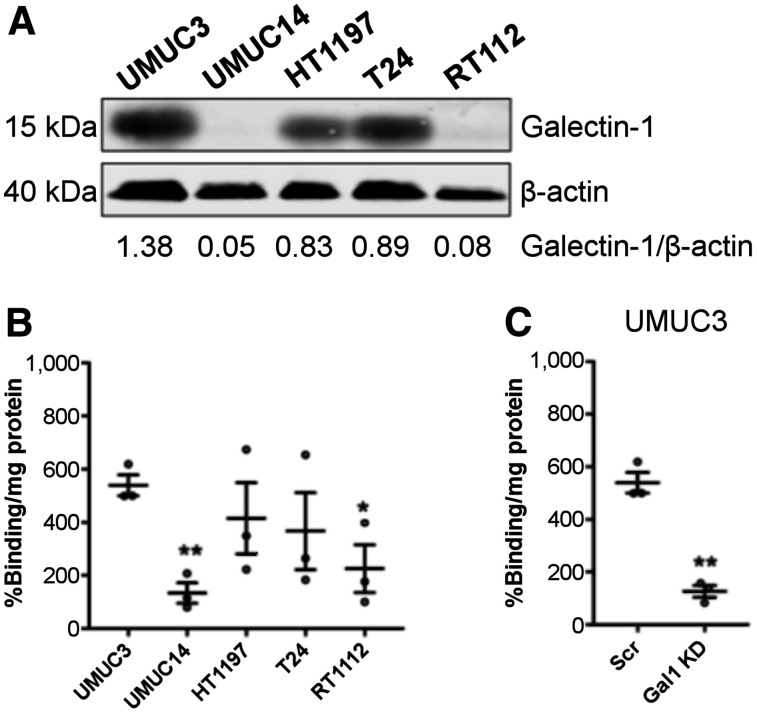
(A) Western blot analysis of galectin-1 in total lysates of UMUC3, UMUC14, HT1197, T24, and RT112 BCa cells. β-actin was used as loading control. (B) ^18^F-labeled galactodendritic unit **4** binding to UMUC3, UMUC14, HT1197, T24, and RT112 BCa cells. *P < 0.05 and **P < 0.001, determined by ANOVA comparing different BCa cells. ANOVA was followed by Tukey post hoc multiple-comparison analysis to determine statistical significance. (C) ^18^F-labeled galactodendritic unit **4** binding to UMUC3 BCa cells before and after galectin-1 knockdown. Data are mean percentage radioactivity/mg of protein ± SEM from 3 independent experiments. *P < 0.05. **P < 0.01 based on Student *t* test and compared with UMUC3 cells. Scr = scrambled small interfering RNA.

To determine the ability of ^18^F-labeled galactodendritic unit **4** to accumulate in BCa cells, galactose radiotracer cell uptake was determined in 5 different BCa cells containing different levels of galectin-1 (Fig. 3A). Notably, binding of ^18^F-labeled galactodendritic unit **4** to BCa cells depends on galectin-1 protein levels. In comparison to UMUC14 and RT112 cells containing low levels of galectin-1, ^18^F-labeled galactodendritic unit **4** displayed a higher cell accumulation in galectin-1–overexpressing UMUC3 cells (Fig. 3B). Additional binding studies demonstrated that small interfering RNA–mediated knockdown of galectin-1 (Supplemental Fig. 10) decreased ^18^F-labeled galactodendritic unit **4** binding to UMUC3 cells (Fig. 3C).

### Imaging of Orthotopic BCa Through ^18^F-Labeled Galactodendritic Unit 4

**FIGURE 4.**
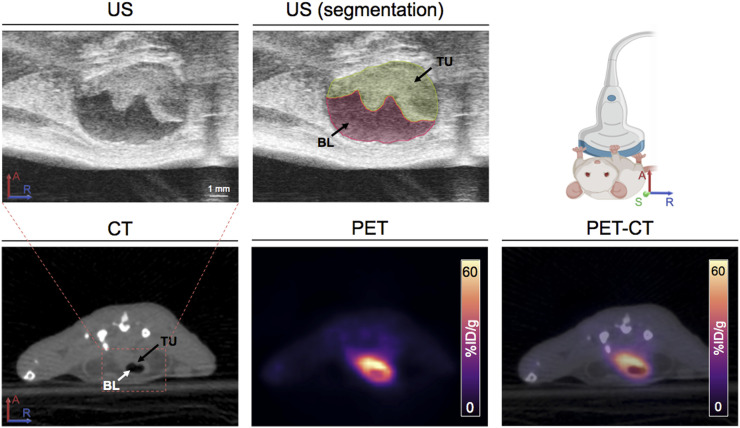
Ultrasound images of murine bladders at 15 d after UMUC3 cells’ implantation in bladder, and representative axial PET/CT images at 1 h after administration of ^18^F-labeled galactodendritic unit **4** in orthotopic UMUC3 bladder tumors. A = anterior; ID = injected dose; BL = bladder; R = right; S = superior; TU = tumor; US = ultrasound.

The ^18^F-labeled galactodendritic unit **4** has high binding to UMUC3 bladder transitional cell carcinoma cells containing a high expression of galectin-1 (Fig. 3) (16,17), and therefore, UMUC3 cells were used to develop an orthotopic BCa model. UMUC3 cells, when seeded onto the mouse urothelium, resemble the clinical condition of non–muscle-invasive bladder cancer (NMIBC) (22). At 15 d after cell inoculation, tumor development was evaluated by ultrasound imaging. UMUC3 cells could be detected growing into murine bladders (Fig. 4) and were used in subsequent experiments.

To determine the biodistribution profile of ^18^F-labeled galactodendritic unit **4,** mice implanted orthotopically with UMUC3 BCa cells were intravenously administered ^18^F-labeled galactodendritic unit **4.** Notably, there was a significant radiotracer accumulation in excretory and metabolic organs (Supplemental Fig. 11), as well as in the bone, as a result of radiotracer catabolism and degradation (Supplemental Fig. 12).

To determine the ability of ^18^F-labeled galactodendritic unit **4** to image BCa, mice implanted orthotopically with UMUC3 bladder tumors were intravesically administered ^18^F-labeled galactodendritic unit **4.** At 30 min after administration, the time at which stability studies demonstrated 75% intact (derived from radiotracer chromatogram) or 50% intact (derived from HPLC chromatogram) ^18^F-labeled galactodendritic unit **4** (Supplemental Fig. 12), the bladder was completely empty and flushed with PBS. Mice without tumors were used as a negative control, receiving an intravesical administration of ^18^F-labeled galactodendritic unit **4.** PET imaging at 1 h after administration showed a significant difference in the uptake of ^18^F-labeled galactodendritic unit **4** between the tumor (Fig. 4) and nontumor (Supplemental Fig. 13) groups. In the tumor group, registration of the PET and CT images showed localization of most remaining activity in the tumor region, revealing targeting of galactodendritic unit **4** to UMUC3 BCa cells. Quantitation of PET images by analyzing regions of interest (3 mice per group) demonstrated a higher SUV_mean_ in tumor mice (Fig. 4) than in the nontumor control group (Supplemental Fig. 13), with values of 43.5 ± 4.2 versus 2.0 ± 0.4, respectively. Additional studies demonstrated that the glucose radiotracer ^18^F-FDG accumulates in UMUC3 orthotopic bladder tumors with an SUV_mean_ of 10.5 ± 2.3 after intravesical administration of the glucose radiotracer (Supplemental Fig. 14). While ^18^F-labeled galactodendritic unit **4** binds to and accumulates in galectin-1–overexpressing UMUC3 bladder tumors (Fig. 4), the glucose radiotracer ^18^F-FDG is reabsorbed after intravesical administration and accumulates in the kidneys and heart (Supplemental Fig. 15).

## DISCUSSION

^18^F-FDG PET imaging in BCa is hampered by ^18^F-FDG renal excretion. Accumulated, renally excreted activity in the bladder interferes with tumor delineation. Additionally, bladder tumor imaging with ^18^F-FDG PET is limited because not all malignant cells take up glucose. The radiotracers ^11^C-choline, ^11^C-methionine, and ^11^C-acetate (all of which have minimal urinary excretion) have been investigated as alternatives to ^18^F-FDG for PET/CT in BCa patients (23). In the context of BCa PET imaging, our group has recently demonstrated in subcutaneous HT1197 bladder tumors the utility of an antibody-based approach using a carbohydrate-antigen 19.9–specific human antibody HuMab-5B1 (MVT-5873) (24).

In previous work, we synthesized a galactose dendritic carbohydrate to be conjugated with porphyrinoids with high affinity to galectin-expressing BCa cells (14,16,17). In preclinical models of BCa, we demonstrated improved tumor binding and efficacy of therapeutic compounds conjugated with such galactose moieties (16,20). In this study, we extended our prior work and radiolabeled dendritic moieties of galactose to generate a PET imaging agent for the diagnosis of galectin-1–overexpressing BCa. We demonstrated by PET imaging that ^18^F-labeled galactodendritic unit **4** specifically accumulates in galectin-1–expressing UMUC3 tumors engrafted in the murine bladder (Figs. 3 and 4). Admittedly, despite the successful *in vivo* demonstration of an effective binding of ^18^F-labeled galactodendritic unit **4** to UMUC3 BCa cells, we were not able to determine the nonspecific binding of the galactose radiotracer, since we did not perform *in vivo* experiments using a BCa cell line containing low levels of galectin-1. As we observed *in vitro* binding of ^18^F-labeled galactodendritic unit **4** to BCa cells containing low levels of galectin-1 (Fig. 3), further studies are necessary to determine the galactose radiotracer ability to bind to other galactose-binding proteins overexpressed in BCa (such as galectin-3). Galectin-1 protein expression is higher in muscle-invasive than non–muscle-invasive BCa (13), and therefore, further studies will determine the ability of ^18^F-labeled galactodendritic unit **4** to differentiate between non–muscle-invasive and muscle-invasive disease.

Carbohydrate radiopharmaceuticals have favorable properties as PET imaging agents: low antigenicity, fast clearance, and good tissue penetration (8). However, their main problems are related to *in vivo* catabolism, accumulation in nontumor tissues, and urinary excretion (8). The ^18^F radionuclide is an ideal short-lived PET isotope (half-life, 109.7 min) for labeling of carbohydrates. Additionally, the ^18^F radionuclide is easily produced in biomedical cyclotrons. Thus, the ^18^F-containing galactodendritic unit **4** was synthesized from the corresponding tosylate precursor **2** in 2 synthetic steps, which were performed *in situ* and produced 45% radiochemical yield (n.d.c.). Similar to ^18^F-FDG, intravenous administration of ^18^F-galactodendritic resulted in renal excretion in the bladder, therefore interfering with tumor delineation. To avoid radioactivity accumulation in the metabolic organs (mainly liver) and excretory organs (e.g., bladder) as a result of ^18^F-galactodendritic unit **4** catabolism and excretion (Supplemental Fig. 11), we decided to perform an intravesical administration of the radiotracer. Intravesical administration of an imaging or therapeutic agent is attractive in BCa because this route is clinically approved for BCa using immunotherapeutic or chemotherapeutic agents and the direct delivery of the agent into the bladder can achieve high concentrations at tumor sites while reducing systemic drug uptake. Intravesical-administration PET/CT with ^18^F-galactodendritic unit **4** was shown to be a powerful tool for orthotopic BCa imaging (Fig. 4), with low accumulation in a nontumor model (Supplemental Fig. 13) and low reabsorption after intravesical administration (Supplemental Fig. 15). ^18^F-FDG was also able to delineate the bladder tumors, suggesting that this glucose radiotracer has potential to image bladder tumors after intravesical administration.

The galactose carbohydrate is almost exclusively catabolized in hepatocytes, and therefore, the ^18^F-labeled galactose has shown potential in the detection of hepatocellular carcinoma (25). The ^18^F-labeled galactodendritic unit **4** is also a potential imaging agent in hepatocellular carcinoma, and further studies are planned in that direction. The ^18^F-labeled galactodendritic unit **4** offers the ability to interact with galectin-1 (Fig. 3), resulting in a high ability to accumulate and image galectin-1–overexpressing bladder tumors (Fig. 4). ^18^F-labeled galactodendritic unit **4** should be able to lead to candidate optimization through further synthetic improvements in its scaffold to enhance stability *in vivo*. From there, possible modifications around the ether bonds could develop more robust galactodendritic structures, resistant to metabolic degradation. In addition to BCa imaging, further studies will explore the potential of intravesical administration of galactodendritic units for BCa therapy using drug-, therapeutic isotope-, photoreactive-bound compounds.

## CONCLUSION

A dendritic galactose moiety was radiolabeled with ^18^F from the corresponding tosylate precursor, resulting in a carbohydrate radiotracer with high affinity to the tumor cells due to its binding with galectin. The ^18^F-labeled dendritic galactose **4** demonstrated high potential as a diagnostic tool for imaging of galectin-1–overexpressing BCa.

## DISCLOSURE

The MSKCC Small Animal Imaging Core Facility and the Radiochemistry and Molecular Imaging Probe Core were supported by NIH grant P30 CA08748. This study was supported in part by the Geoffrey Beene Cancer Research Center of MSKCC (Jason Lewis), NIH NCI R35 CA232130 (Jason Lewis), the Mr. William H. and Mrs. Alice Goodwin and the Commonwealth Foundation for Cancer Research, the Center for Experimental Therapeutics of Memorial Sloan Kettering Cancer Center, and a Tow Foundation Postdoctoral Fellowship (Patricia Pereira), as well as by Fundação para a Ciência e a Tecnologia (FCT, Portugal), the European Union, QREN, FEDER through Programa Operacional Factores de Competitividade (COMPETE), QOPNA (FCT UID/QUI/00062/2019), and CQE (FCT UID/QUI/0100/2019) research units, which are financed by national funds through the FCT/MEC and, when appropriate, cofinanced by FEDER under the PT2020 Partnership Agreement. The research contract of Flávio Figueira (REF-168-89-ARH/2018) is funded by national funds (OE) through FCT. No other potential conflict of interest relevant to this article was reported.

**KEY POINTS**

**QUESTION:** Can galectin-expressing BCa be imaged with a new ^18^F-labeled galactodendritic unit?
**PERTINENT FINDINGS:** An ^18^F-labeled first-generation galactodendritic unit was obtained from its tosylate precursor. Intravesical administration of ^18^F-labeled galactodendritic unit allowed specific accumulation of the carbohydrate radiotracer in orthotopic bladder tumors when imaged with PET. The ^18^F-labeled galactodendritic unit was not found to accumulate in nontumor murine bladders.
**IMPLICATIONS FOR PATIENT CARE:** The ^18^F-labeled galactodendritic unit and similar analogs may be exploitable for PET imaging, as a complementary diagnostic technique, to detect galectin-1–overexpressing bladder tumors.
