# Supplementary material for: PET/CT Imaging with an 18F-Labeled Galactodendritic Unit in a Galectin-1–Overexpressing Orthotopic Bladder Cancer Model
Source: J Nucl Med. 2020 Sep;61(9):1369–75. doi: 10.2967/jnumed.119.236430 (PMC7456169; doi:10.2967/jnumed.119.236430)
Supplement: Supplementary file 1 [file jnm236430SupplementaryData.pdf]

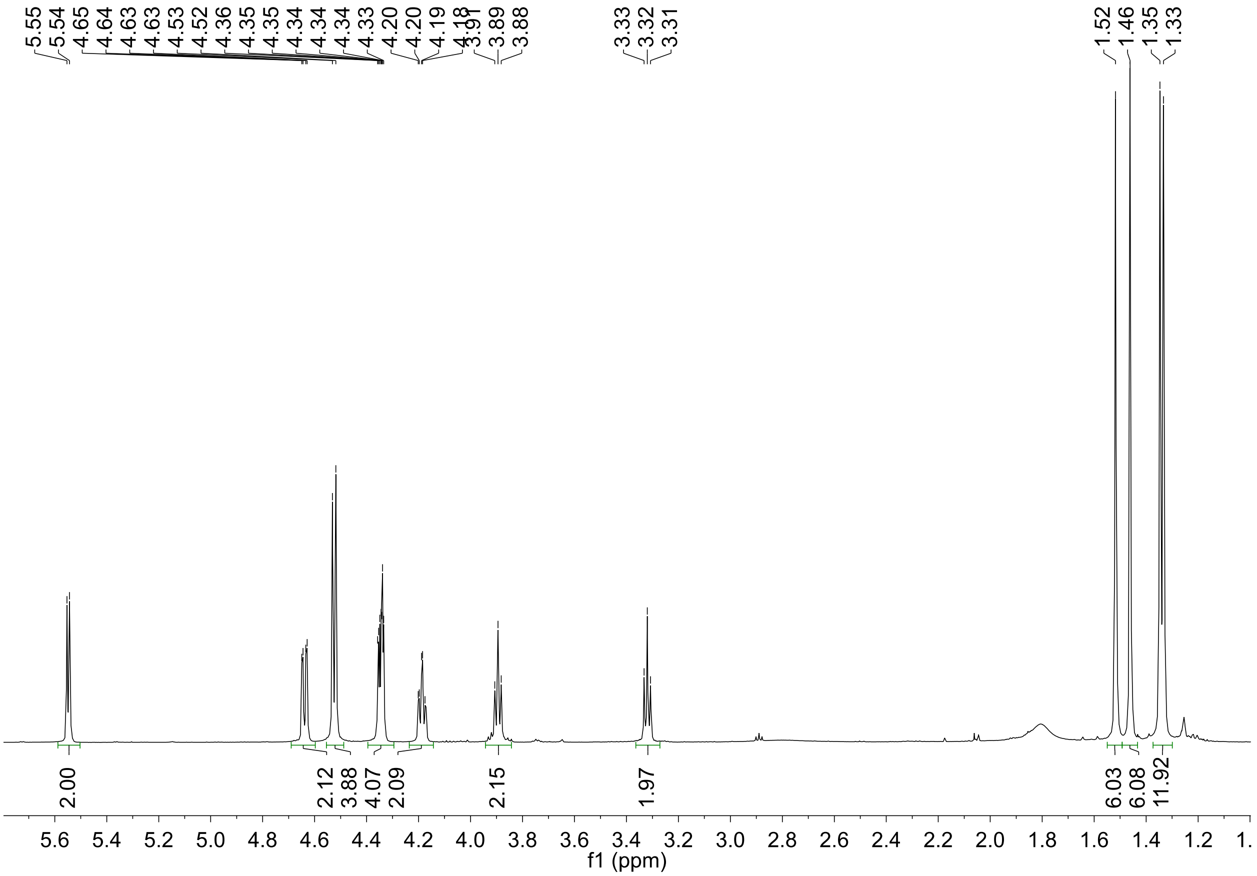

Supplemental Figure 1. ^{1}H NMR spectra of compound **1.** Compound **1** was dissolved in chloroform-*d* and ^{1}H NMR spectra acquired at 500 MHz. The full interpretation of the data is in the results section of the manuscript.

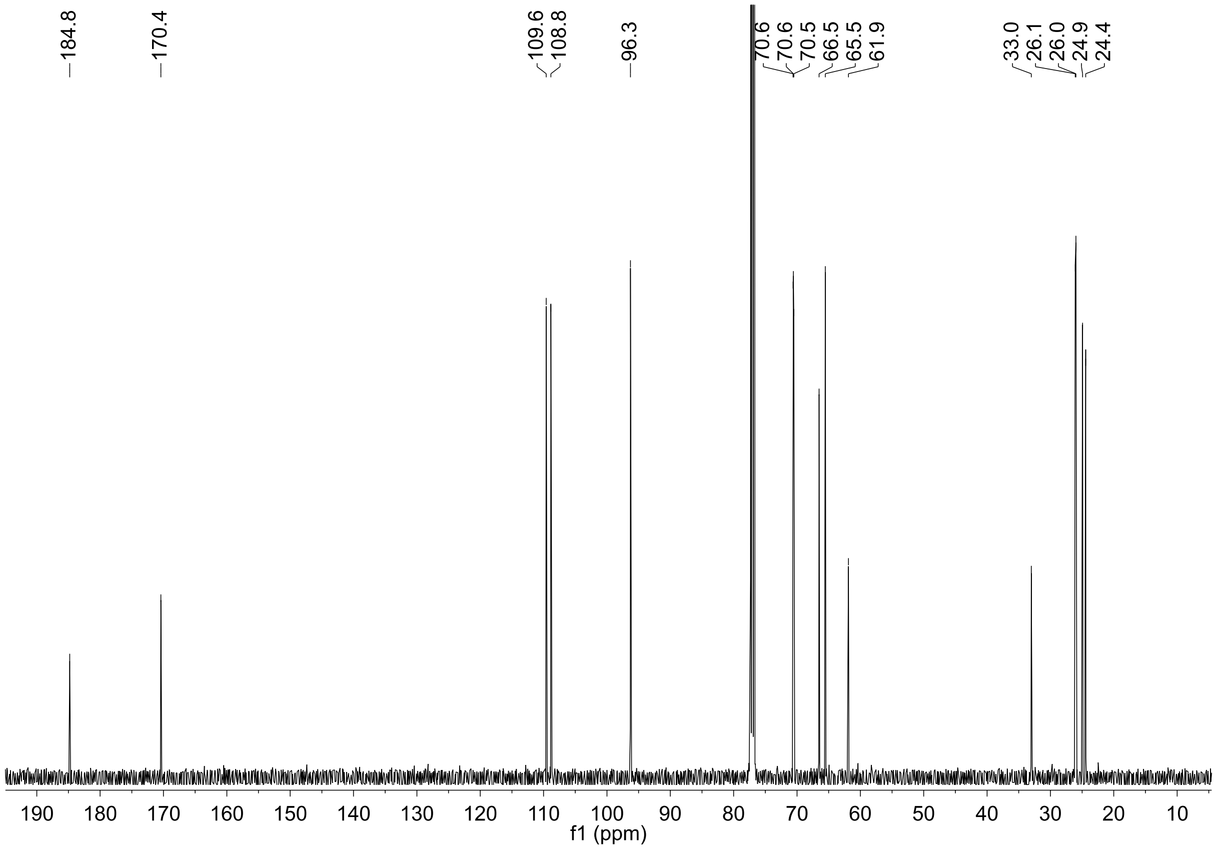

Supplemental Figure 2. ^{13}C NMR spectra of compound **1.** Compound **1** was dissolved in chloroform-*d* and ^{1}H NMR spectra acquired at 500 MHz. The full interpretation of the data is in the results section of the manuscript.

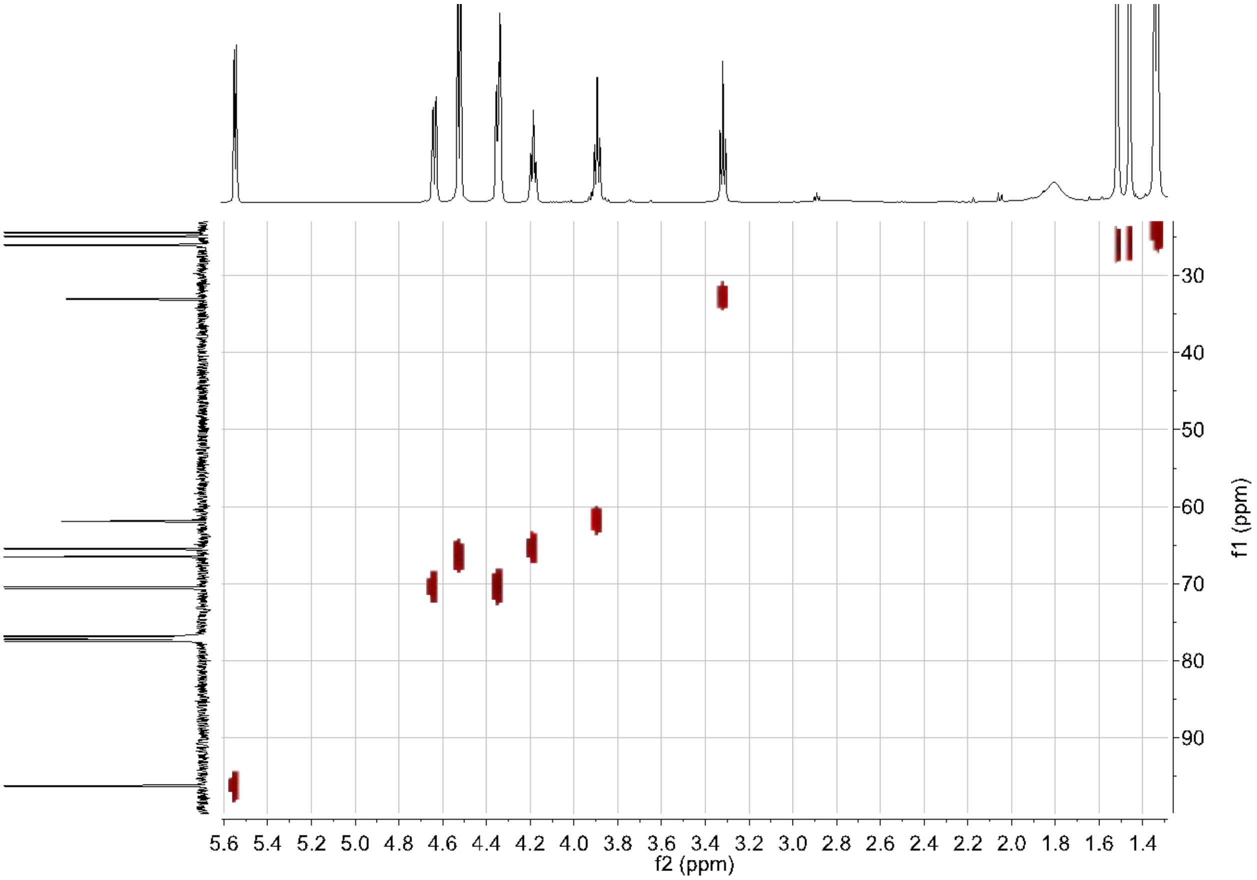

Supplemental Figure 3. Heteronuclear Single Quantum Coherence (HSQC) spectra of compound **1** to determine proton-carbon single bond correlations. Protons lie along the observed x-axis and carbons lie along the observed y-axis. Full interpretations of the data allowed for the full C-H structure determination of the compound reported in the results section of the manuscript.

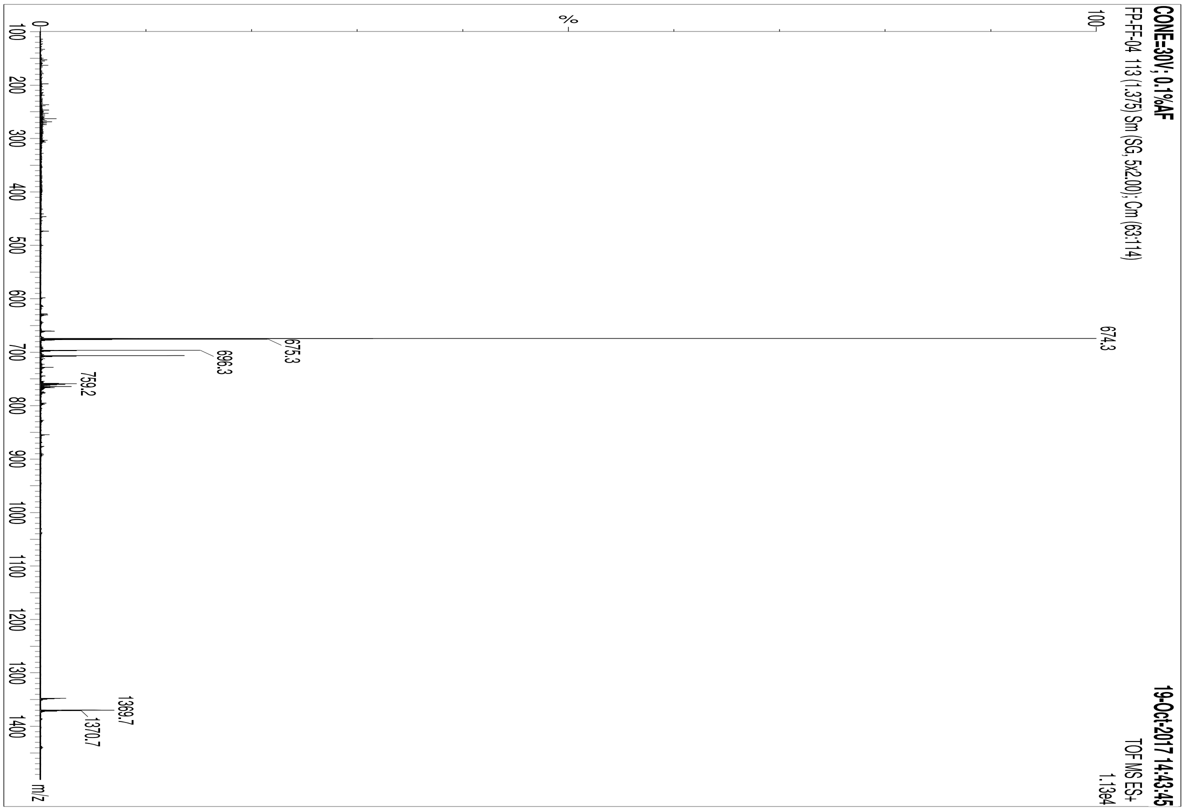

Supplemental Figure 4. Electrospray ionization mass spectrometry (ESI-MS) spectra of compound **1.** Calculated mass to charge ratio (*m/z*) for compound **1** was 673.25 and mass found was 674.3 $[M + H]^+$.

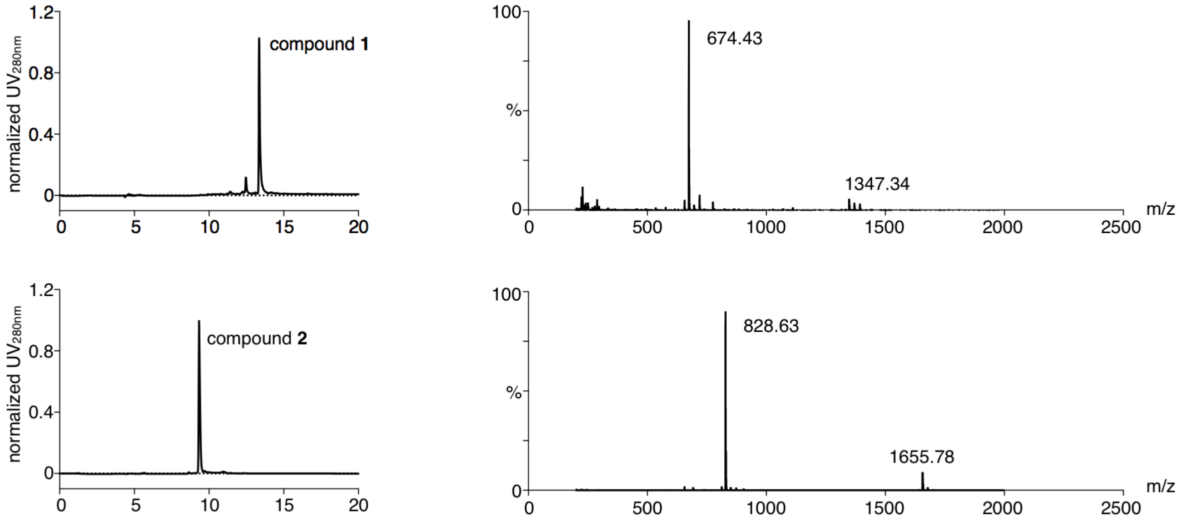

Supplemental Figure 5. HPLC chromatogram (*left*) ESI-MS spectra (*right*) of compound **1** and compound **2.** Calculated mass to charge ratio (*m/z*) for compound **2** was 827.26 and mass found 828.63 $[M + H]^+$.

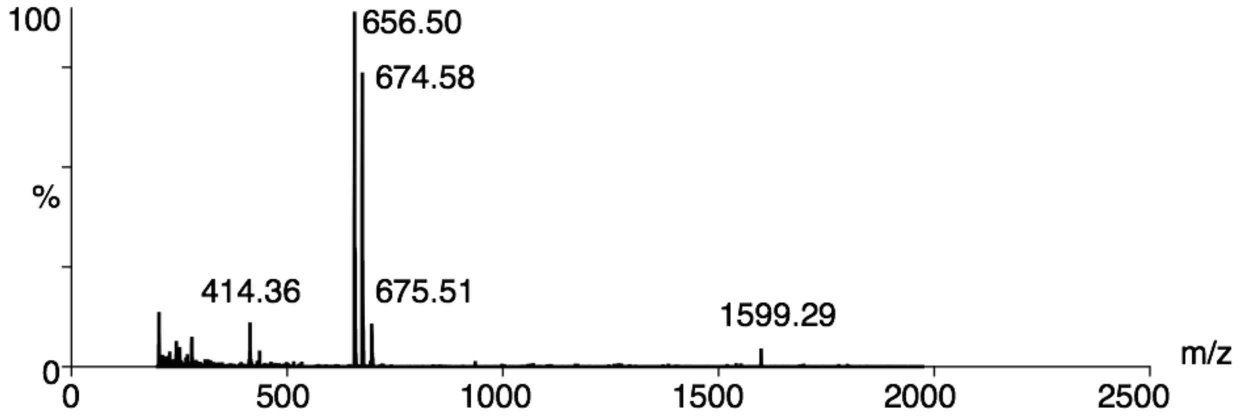

Supplemental Figure 6. ESI-MS spectra of compound **3.** Calculated mass to charge ratio (*m/z*) for compound **3** was 656.25 and mass found 656.50 $[M + H]^+$.

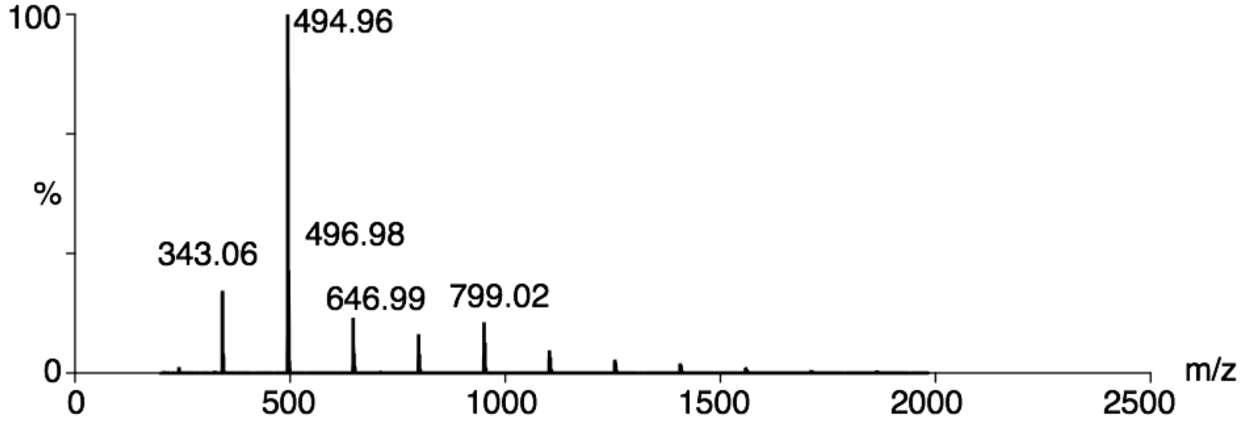

Supplemental Figure 7. ESI-MS spectra of compound **4**. Calculated mass to charge ratio (*m/z*) for compound **3** was 496.12 and mass found 496.98 $[M + H]^+$.

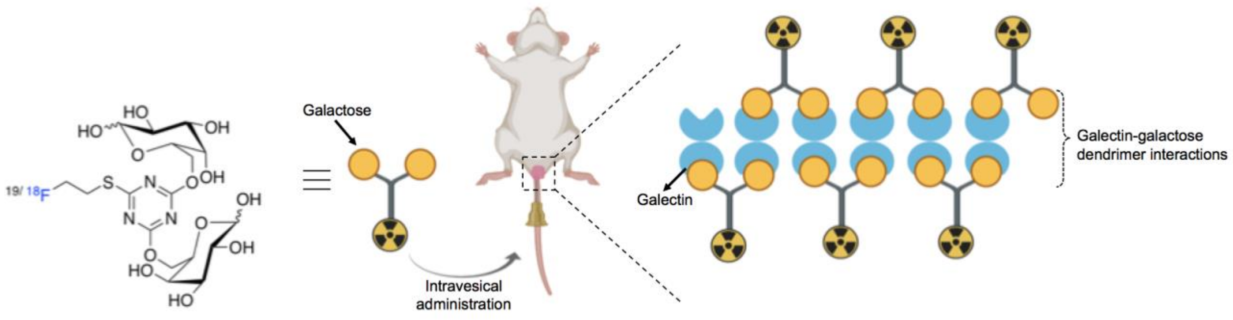

Supplemental Figure 8. The F-18 containing galactodendritic unit **4** was administered via intravesical injections into the bladder of mice bearing orthotopic UMUC3 bladder cancer cells. The galactose dendritic moieties in ^{18}F-labeled galactodendritic unit **4** interact with galectin-1 at the tumor cells, allowing BCa PET imaging.

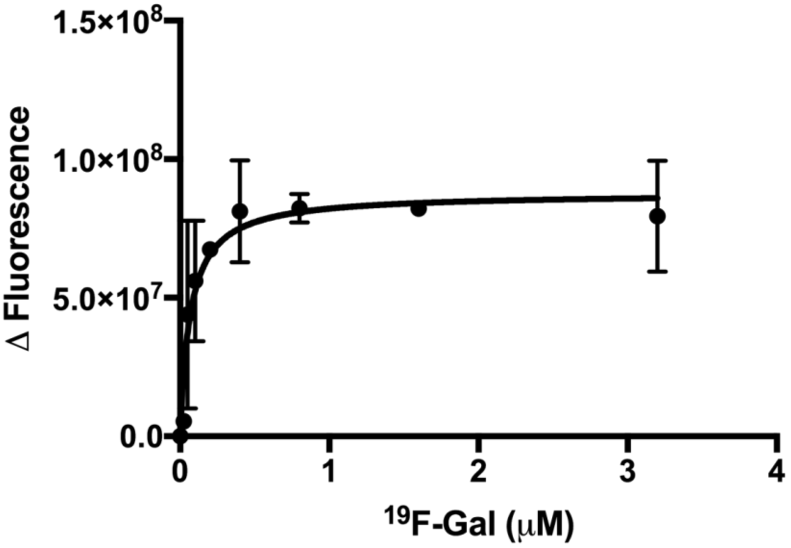

Supplemental Figure 9. Fluorescence variation on the emission spectrum of 2 μM galectin-1 protein after the addition of [^{19}F]compound **4**. Data are means ± S.E.M. of three independent experiments.

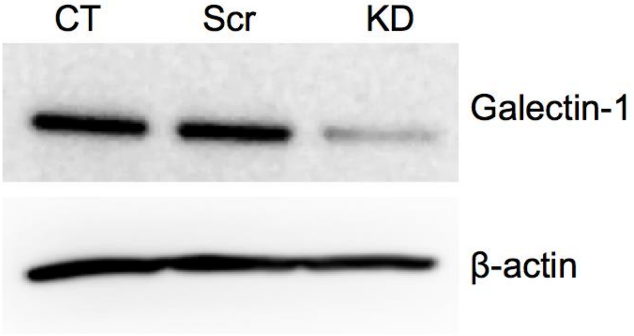

Supplemental Figure 10. Western blot analysis of galectin-1 in the total lysates of UMUC3 before and after knockdown using siRNA. Scr, scrambled siRNA.

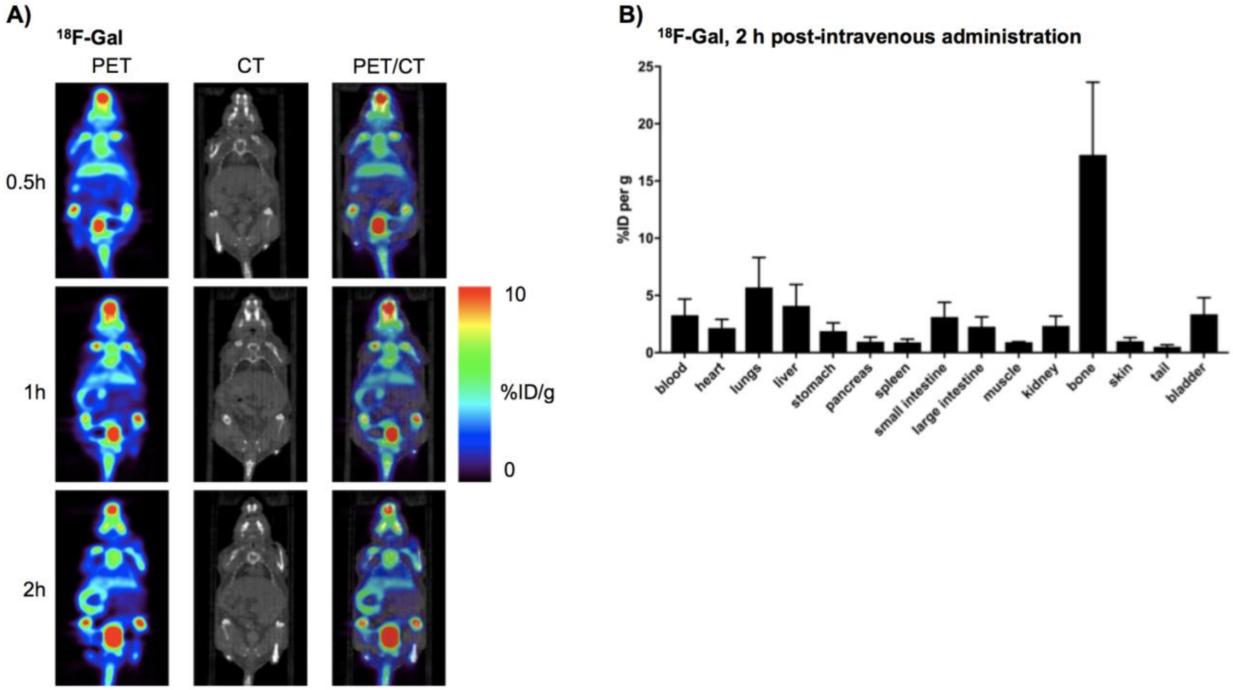

Supplemental Figure 11. (A) Representative coronal PET images and (**B**) biodistribution data of ^{18}F-labeled galactodendritic unit **4** in athymic nude mice bearing orthotopic UMUC3 bladder tumors. Mice were intravenous administrated ^{18}F-labeled galactodendritic unit **4** (2.9 – 3.3 MBq) and PET/CT images acquired at 0.5, 1, and 2 h post-administration of the galactose radiotracer. Biodistribution was performed at 2 h post-injection of ^{18}F-labeled galactodendritic unit **4** (Bars, n = 4 mice per group, mean ± S.E.M). CT, Computer Tomography; PET, Positron Emission Tomography; %ID/g, percentage of injected dose per gram of tissue.

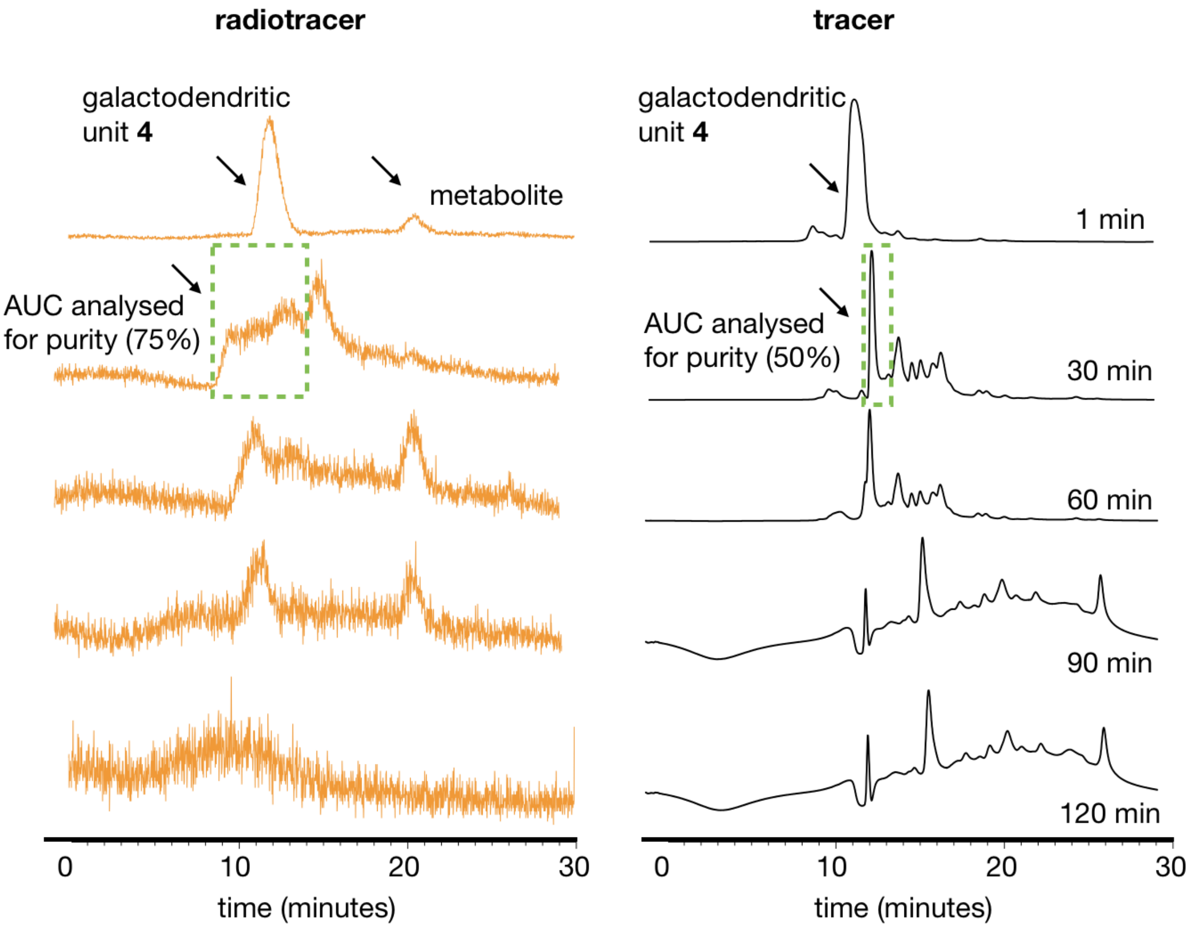

Supplemental Figure 12. Stability of ^{18}F-labeled galactodendritic unit **4** in saline sample containing 10.5% (v/v) mice urine. (**left**) Radiotracer chromatogram of ^{18}F-labeled galactodendritic unit **4** in a saline sample containing mice urine at various time-points post-incubation (1 min, 30 min, 60 min, 90 min, and 120 min). (**right**) Corresponding HPLC chromatogram (280 nm) of ^{18}F-labeled galactodendritic unit **4** in a saline sample containing mice urine at various time-points post-incubation (1 min, 30 min, 60 min, 90 min, and 120 min). AUC, area under the curve.

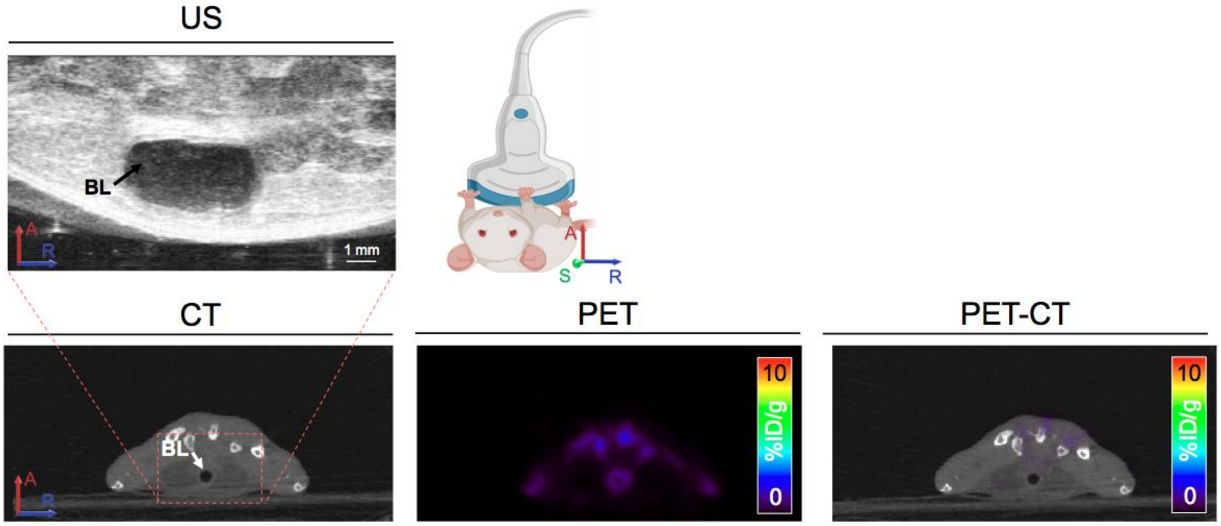

Supplemental Figure 13. Top panel, Ultrasound image of murine bladders of non-tumor bearing mice. **Lower panel**, Representative axial PET images at 1 h after administration of ^{18}F-labeled galactodendritic unit **4** in athymic nude mice. Mice were intravesical administrated ^{18}F-labeled galactodendritic unit **4** (14.7 – 15.3 MBq,), the bladder was flushed with PBS, and PET/CT images were acquired at 1 h post-administration of the galactose radiotracer. US, ultrasound; BL, bladder; TU, tumor; CT, Computer Tomography; PET, Positron Emission Tomography.

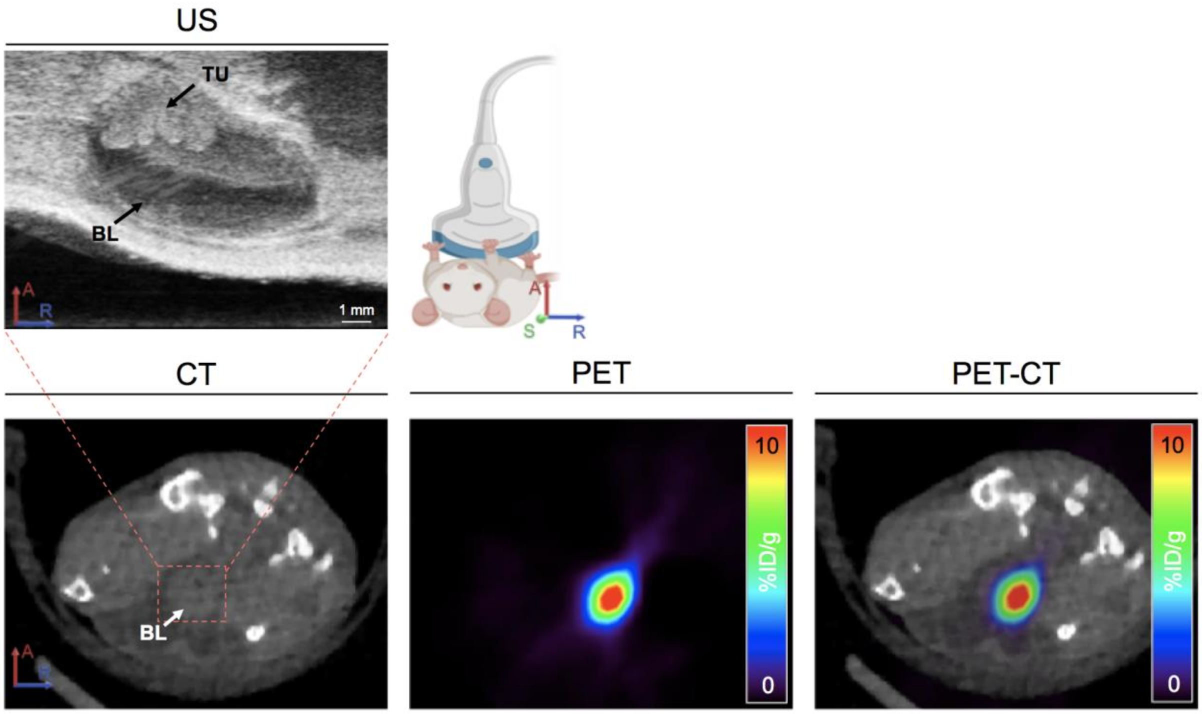

Supplemental Figure 14. Top panel, Ultrasound images of murine bladders at 15 days after UMUC3 cells' implantation in the bladder. **Lower panel**, Representative axial PET images at 1 h after administration of ^{18}F-FDG in athymic nude mice bearing orthotopic UMUC3 bladder tumors. Mice were intravesical administrated ^{18}F-FDG (14.7 – 15.3 MBq), the bladder flushed with PBS, and PET/CT images acquired at 1 h post-administration of the glucose radiotracer. US, ultrasound; BL, bladder; TU, tumor; CT, Computer Tomography; PET, Positron Emission Tomography.

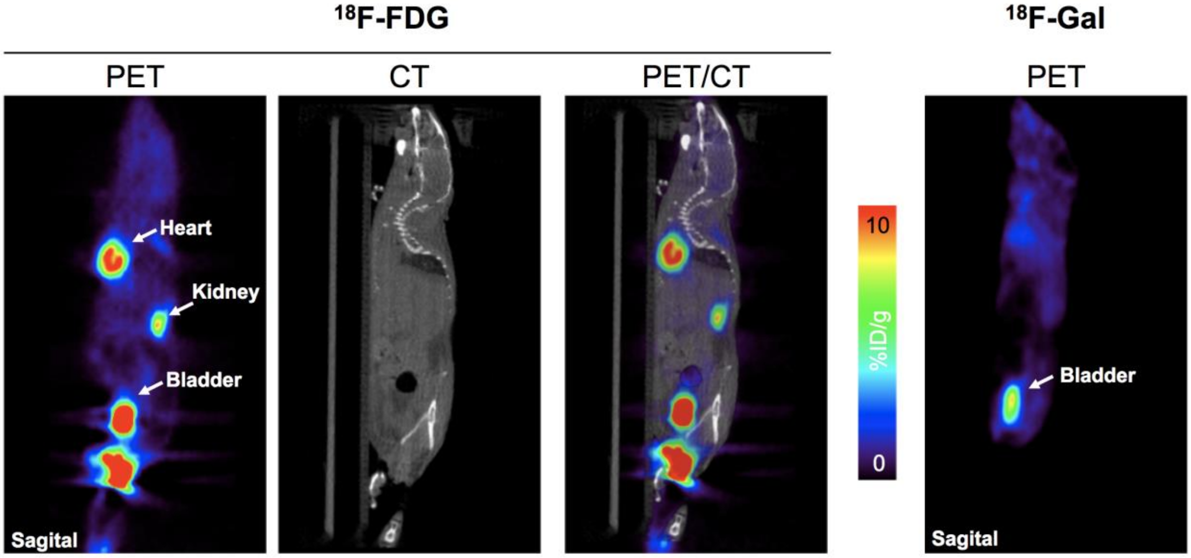

Supplemental Figure 15. Representative sagittal PET images of (**left**) ^{18}F-FDG and (**right**) ^{18}F-labeled galactodendritic unit **4** in athymic nude mice bearing orthotopic UMUC3 bladder tumors. Mice were intravesical administrated ^{18}F-FDG or ^{18}F-labeled galactodendritic unit **4** (14.7 – 15.3 MBq), the bladder was flushed with PBS, and PET/CT images were acquired at 1 h post-administration of the glucose or galactose radiotracer. CT, Computer Tomography; PET, Positron Emission Tomography.
